# Speech analysis and speech emotion recognition in mental disease: a scoping review

**DOI:** 10.3389/fpsyg.2025.1645860

**Published:** 2025-11-06

**Authors:** Clara Lombardo, Giulia Esposito, Silvia Carbone, Salvatore Serrano, Carmela Mento

**Affiliations:** 1 Department “Scienze della Salute”, University of Catanzaro, Catanzaro, Italy; 2 Department of Engineering, University of Messina, Messina, Italy; 3 Political and Legal Sciences Department, University of Messina, Messina, Italy; 4 Department of Biomedical and Dental Sciences and Morphofunctional Imaging, University of Messina, Messina, Italy

**Keywords:** speech analysis, acoustic features, speech emotion recognition, mental disorders, schizophrenia, depression

## Abstract

**Background:**

Mental disorders have a significant impact on many areas of people’s life, particularly on affective regulation; thus, there is a growing need to find disease-specific biomarkers to improve early diagnosis. Recently, machine learning technology using speech analysis proved to be a promising field that could aid mental health assessments. Furthermore, as prosodic expressions of emotions are altered in many psychiatric conditions, some studies successfully employed a speech emotion recognition model (SER) to identify mental diseases. The aim of this paper is to discuss the utilization of speech analysis in diagnosis of mental disorders, with a focus on studies using SER system to detect mental illness.

**Method:**

We searched PubMed, Scopus and Google Scholar for papers published from 2014 to 2024. We conducted a preliminary search, which revealed papers on the topic. Finally, 12 studies met the inclusion criteria and were included in the review.

**Results:**

Findings confirmed the efficacy of speech analysis in distinguishing between patients from healthy subjects; moreover, the examined studies underlined that some mental illnesses are associated with specific voice patterns. Furthermore, results from studies employing speech emotion recognition system to detect mental disorders showed that emotions can be successfully used as an intermediary step for mental diseases detection, particularly for mood disorders.

**Conclusion:**

These findings support the implementing of speech signals analysis in mental health assessment: it is an accessible and non-invasive method which can provide earlier diagnosis and a higher treatment personalization.

## 1 Introduction

A psychiatric disorder is a mental or behavioral pattern that influences emotional regulation, behavior and cognition, causing a significant impairment in several areas of people’s life, such as the functioning capacity at work and with their families (Lalitha et al., 2021). In recent years, especially during the Covid-19 pandemic, there has been a significant increase in people affected by a mental disorder, with a consequent high impact on emotional life and affective regulation: about 970 million people in the world are currently suffering from a mental disorder and the number is expected to grow in the future (Cansel et al., 2023). To now, there is still a lack of biomarkers and individualized treatment guidelines for mental illnesses (Chen et al., 2022). In this regard, precision medicine is emerging in psychiatry as an innovative approach to improve the diagnosis and treatment of mental disorders, through a higher individualization of care and attention to the unique characteristics of each patient (Manchia et al., 2020). Machine learning technology seems to be a promising field in mental health assessments: it may indeed be useful in screening of at-risk patients, improve the detection of disorder-specific features, allow to plan more efficient treatments and enable more real-time monitoring of psychiatric disorders (Low et al., 2020; Siena et al., 2020).

In particular, the language can be considered as a window into the mind (Koops et al., 2023): people convey emotions, thoughts and motivations through speech (Zhang et al., 2024). If the speech content is easily masked by people, features such as speed, energy and pitch variation in speech cannot be controlled. Therefore, vocal-acoustic cues allow to get an objective measurement of mental illness (Patil and Wadhai, 2021). Many studies have demonstrated that acoustic parameters can be used as valid biomarkers for the early diagnosis of mental disorders (Pan et al., 2019; Cummins et al., 2015). The most common acoustic features analyzed are the spectral features, related to the energy or the spectral flatness, the prosodic features describing the speech intonation, rhythm and rate, the temporal characteristics (e.g., utterance duration, duration and number of pauses) and the cepstral features that are commonly used in speech recognition for their high performance in describing the variation of low frequencies of the signal (Jiang et al., 2018; Teixeira et al., 2023). These features can reflect emotional arousal and expressiveness. Specifically, the ones referred to prosody give information about speech emotional tone and dynamics of speech. For example, Hashim et al. (2017) noticed that acoustic speech signals alterations characterizing spectrum and timing are useful to examine depressive symptoms levels and treatment effectiveness. Other studies have instead shown that depression was associated with changes in prosody, such as an overall speech rate (Alghowinem et al., 2013; Wang et al., 2021), and changes in speech spectrum, like the decrease in the sub-band energy variance (Cummins et al., 2015). Furthermore, a recent meta-analysis on schizophrenic acoustic patterns showed that patients presented reduced speech rate and pitch variability (Parola et al., 2018).

Negative emotions such as sadness, anger and fear are indicator of mental disorders (Lalitha and Tripathi, 2016): for this reason, another promising approach to diagnosis of mental health conditions comes from Speech Emotion Recognition (SER), a system which provides an extraction of the speakers’ emotional states from their speech signals (Hashem et al., 2023; Kerkeni et al., 2019). It has been employed in detecting different mental illnesses, such as post-traumatic stress disorder (PTSD; Pathan et al., 2023) and depression (Mar and Pa, 2019). In particular, SER model utilization for depression prediction is supported by findings about the inhibition of prosodic emotional expression in depressive conditions, but also by experimental studies connecting positively SER and depression detection models (Stasak et al., 2016). Harati et al. (2018) carried out a computational speech analysis for classifying depression severity applying Deep Neural Network (DNN) model to audio recordings of patients with Major Depressive Disorder. Participants in this research are evaluated weekly for 8 months, starting before Deep Brain Stimulation (DBS) and throughout the first 6 months of DBS surgery; two clinical phases are therefore considered for the speech analysis: depressed and improved. This approach successfully classified the two phases of DBS treatment with an AUC of 0.80. Furthermore, Bhavya et al. (2023) proposed a new computational methodology to detect different emotions and depression; specifically, they built a dataset for depression-related data using audio samples from the DAIC-WOZ depression dataset and the RAVDESS dataset, which includes a wide spectrum of emotions conveyed by speakers of both genders. This method proved to be useful to recognize depressive symptoms.

While several reviews have analyzed general aspects regarding the diagnostic use of speech as biomarker for the early diagnosis of mental disorders (Low et al., 2020), few have particularly considered studies employing speech emotion recognition (SER) systems. This shows that there is a gap in synthesizing results specifically emerging from diagnostic studies employing SER. Therefore, the aim of this work is to supply an updated analysis of literature on acoustic features used as objective indicators for the diagnosis of mental disorders, with a focus on studies using speech emotion recognition system. This is in order to confirm the effectiveness of this approach in mental health assessments.

## 2 Materials and methods

**Figure 1:**
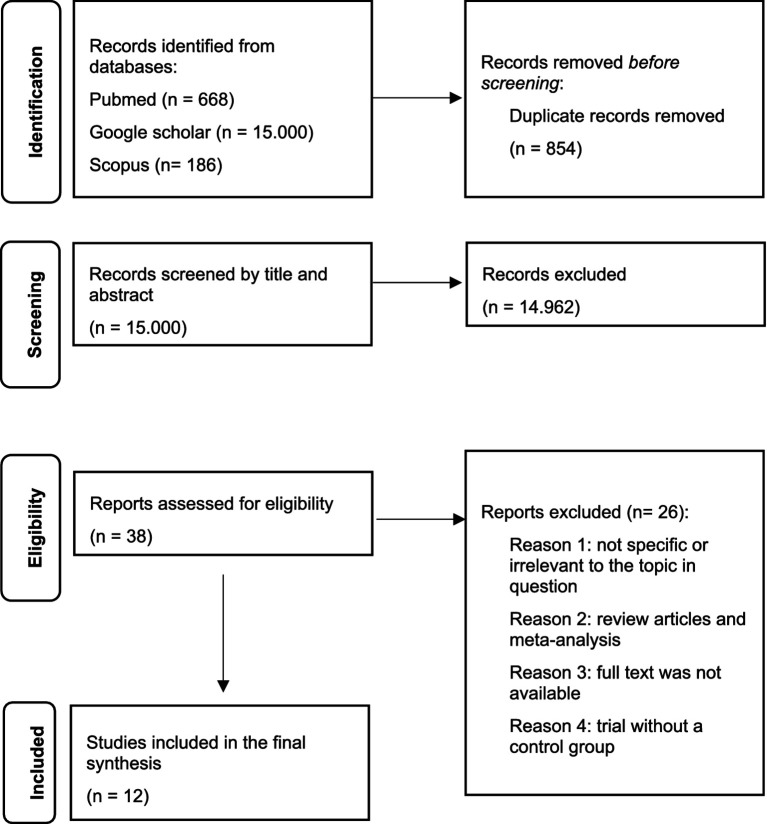
PRISMA-ScR flow diagram.

This scoping review was conducted and reported in accordance with the PRISMA extension for Scoping Reviews (PRISMA-ScR) guidelines (Tricco et al., 2018). The PRISMA-ScR flow diagram (Figure 1) illustrates the study selection process.

### 2.1 Information sources and search strategy

We searched PubMed, Scopus and Google Scholar, for papers published from January 1, 2014 to November 1, 2024, with combinations of the following search terms: *“Speech analysis OR speech emotion recognition OR acoustic analysis OR acoustic features AND mental disorders AND schizophrenia AND depression AND bipolar disorder.”*

### 2.2 Data extraction

We conducted a preliminary search, which revealed papers on the topic. Articles were included in the review according to the following inclusion criteria: English language, only empirical studies (e.g., observational, non-randomized experimental and machine learning classification designs), studies involving clinical populations with mental disorders, studies that involved quantitative and/or qualitative assessments of the variables considered. Books, meta-analyses, and reviews were excluded; non-empirical studies and studies that did not involve quantitative and/or qualitative assessments of the variables were also excluded.

### 2.3 Data synthesis

We found 15.854 articles. Of these, 854 were removed before screening since they were duplicates. At the first screening conducted by title and abstract, 14.962 studies were excluded. After the second screening conducted by full-text examination of 38 papers, 26 articles were excluded because they were reviews, meta-analysis, not specific, irrelevant for the topic, because full text was not available or because the trial did not present a control group. Finally, 12 studies met the inclusion criteria and were included in the review. Due to the high heterogeneity of the studies, a qualitative data analysis was conducted instead of a quantitative meta-analysis. The annexed table summarizes the selected articles (Table 1), whereas the annexed flow diagram (Figure 1) summarizes the selection process.

**Table 1:** Main results of included studies.

Authors	Aim	Sample	Materials and measures	Speech/ emotion analysis methods	Results
Martínez-Sánchez et al. (2015)	Quantify the deficits in expressive prosody in schizophrenia and evaluate its discriminatory power between groups	*N* = 80 −45 patients with schizophrenia (M = 39.49, SD = 10.89; 71.1% male) −35 controls (M = 35.34, SD = 10.48; 62.9% male)	Brief Psychiatric Rating Scale (BPRS) Professional Fostex FR-2LE recorder Acoustic voice analysis 5.1.42 Praat program	Acoustic voice analysis 5.1.42 Praat program for the extraction of different parameters (e.g., pitch, duration, temporal variations and pauses) related to expressive prosody.	Schizophrenic patients showed significantly more pauses (*p* < 0.001), less pitch variability in speech (*p* < 0.05), fewer variations in syllable timing (*p* < 0.001) and they were slower (*p* < 0.001) than control subjects. Signal processing algorithms applied to speech were shown an accuracy of 93.8% in distinguishing patients from healthy controls.
Scherer et al., 2015	Explore vowel space, a measure of frequency range, extracted from conversational Speech, and its relationship to self-reported symptoms of depression and post-traumatic stress disorder (PTSD)	*N* = 253 Depression group = 47 (33 male and 14 female) PTSD group = 88 (58 male and 30 female) No depression group = 205 (153 male and 52 female) No PTSD group = 165 (128 male and 37 female)	PTSD Checklist-Civilian version (PCL-C) Patient Health Questionnaire-Depression 9 (PHQ-9) COVAREP toolbox for the processing of the speech signals	COVAREP toolbox for the processing of the speech signals (vowel space, formants, pitch, energy)	Results showed a significantly reduced vowel space in subjects that scored positively on the questionnaires of PTSD (PTSD *M* = 0.51, non-PTSD *M* = 0.56, *t*(251) = 2.55, *p* = 0.01 Hedges’ *g* = −0.34) and depression (depressed *M* = 0.49, non-depressed *M* = 0.55, *t* (251) = 2.69, *p* = 0.008, Hedges’ *g* = −0.43).
Chakraborty et al., 2018	Employ low-level speech signals in the distinction of patients with schizophrenia from healthy individuals.	*N* = 78 52 patients with Schizophrenia −26 healthy controls	Brief Assessment of Cognition (BAC) Semi-structured clinical interview NSA-16 OpenSMILE ‘emobase’ to recognize emotion from acoustic signals.	OpenSMILE “emobase” to extract low-level prosodic features (e.g., intonation, energy, duration)	The objective openSMILE acoustic signals can be reliably used to distinguish between the patient and controls with an accuracy of 79–86%.
Stolar et al., 2018	Detect depression with a clinical database of adolescents interacting with a parent.	*N* = 63 Depressed patients = 29 (5 male and 24 female) Healthy controls = 34 (10 male and 24 female)	Voice activity detector (VAD) to extract voiced speech segments	Voice activity detector (VAD) for the analysis of spectral parameters of speech (e.g., flux, centroids, formants and optimized spectral roll-off)	The proposed optimized feature set achieved an average depression detection accuracy of 82.2% for males and 70.5% for females. Among acoustic spectral features, the optimized spectral roll-off set is the most effective.
Tahir et al., 2019	Explore non-verbal speech signals as objective measures of negative symptoms of schizophrenia, studying the correlation with the subjective ratings of negative symptoms on a clinical scale.	*N* = 80 −54 patients with schizophrenia −26 healthy controls	The Structured Clinical Interview for DSM-IV (SCID) −16-item Negative Symptom Assessment (NSA-16)	Automatic extraction of non-verbal speech cues (e.g., pause duration, speech ratio, turn-taking and prosodic variability) through speech segmentation algorithms in Matlab.	The study allows to distinguish healthy and patients using non-verbal speech features (conversational and prosody related cues) with 81.3% accuracy.
He et al., 2020	Evaluate an automatic system for detecting the negative symptoms of patients with schizophrenia based on speech signal processing.	*N* = 56 −28 patients with schizophrenia (18 females and 10 males) −28 healthy controls (18 females and 10 males)	Psychotic Disorders Severity Scale Reading three texts to express emotions and analyze the associated speech signals. Decision tree for features classification	Automatic acoustic signal processing system based on three features: SDVV (speech intensity), SSDL (spectral difference), QEVA (tone variation).	The most promising feature is the SDVV feature: it achieves an accuracy of more than 85% in the detection of schizophrenic patients’ speech in each emotional state. The combination of three acoustic features (SSDL, QEVA, SDVV) achieved a high level of accuracy (98.2%, with an AUC value of 98%) in discrimination of schizophrenic patients and controls.
Lee et al., 2021	Develop a voice-based screening test for depression using vocal acoustic features of elderly people, for males and females.	*N* = 204 Depressed patients = 61 Healthy controls = 143	Mini International Neuropsychiatric Interview (MINI-K) Korean version of the Consortium to Establish a Registry for Alzheimer’s Disease Assessment Packet Clinical Assessment Battery (CERAD-K-C) Digit Span Test Frontal Assessment Battery Korean version of the geriatric depression scale (GDS-KR) Mood-inducing sentences (MIS) OpenSMILE v2.1.0 for speech analysis	OpenSMILE v2.1.0 to extract spectral, prosodic and energy features with AVEC 2013 and eGeMAPS sets	Acoustic features showing significant discriminatory performances are spectral and energy-related features for males (sensitivity 0.95, specificity 0.88, and accuracy 0.86) and prosody-related features for females (sensitivity 0.73, specificity 0.86, and accuracy 0.77).
Patil and Wadhai, 2021	Use acoustic features extracted from the spontaneous speech samples of the volunteers to detect depression.	*N* = 129 Depressed patients = 54 Healthy controls = 75	Patient Health Questionnaire (PHQ-9) Depression Inventory (BDI) scale Praat 6.0 for speech analysis	Praat v6.0 to extract parameters such as MFCC, pitch, jitter, shimmer, and energy SVM, Random Forest, GMM classifiers for depression detection	Speech features like MFCC, pitch, jitter, shimmer and energy can be used as a reliable biomarker for depression detection.
Hansen et al., 2022	Explore a method to allow a clinical evaluation of depression and remission from acoustic speech	*N* = 82 Healthy controls = 42 Patients group = 40 individuals with first-episode major depressive disorder (MDD) Patients in remission = 25	Hamilton Rating Scale for Depression, to evaluate depression severity and remission Audio recordings of the Indiana Psychiatric Illness Interview A gradient boosted decision tree model was trained to predict the probability of sounding happy or sad and combined in a Mixture of Experts (MoE) architecture for ensemble prediction	A gradient boosted decision tree model trained to predict the probability of sounding happy or sad and combined in a Mixture of Experts (MoE) architecture for ensemble prediction	Patients with depression have a probability of sounding sad (theta) of 0.70 (95% CI: 0.38, 0.90); patients in remission have a theta of 0.25 (95% CI: 0.07, 0.58); healthy controls at visit 1 have a theta of 0.23 (95% CI: 0.10, 0.47), and at visit 2 have a theta of 0.22 (95% CI: 0.07, 0.58). SER model allows to distinguish between depressed patients and healthy controls, achieving an AUC of 0.71.
Rejaibi et al., 2022	Present a deep Recurrent Neural Network-based framework to detect depression and to predict its severity level from speech.	*N* = 189 Depressed patients = 56 (25 male and 31 female) Healthy controls = 133 (77 male and 56 female)	Patient Health Questionnaire of eight questions (PHQ-8) DAIC-WOZ depression dataset	DAIC-WOZ depression dataset (extraction of low-level and high-level MFCC features from clinical audio) RNN model for depression detection and PHQ-8 score prediction	The proposed approach obtained an accuracy of 76.27% in detecting depression. MFCC based high-level features give relevant information about depression.
Wanderley Espinola et al., 2022	Present a methodology to support the diagnosis of schizophrenia, major depressive disorder, bipolar disorder, and generalized anxiety disorder using vocal acoustic analysis and machine learning.	*N* = 78 Depression group: 28 (17 males) Schizophrenia group: 21 (12 males) Bipolar Disorder group: 14 Generalized anxiety Disorder group: 4 Control group: 12 (7 males)	Depression: HAM-D Schizophrenia: BPRS Bipolar disorder: YRMS GAD: GAD-7 Control group: SRQ-20 Acquisition of voice samples: Tascam™ 16-bit linear PCM recorder Audio editing: Audacity™ audio software Feature extraction: GNU Octave™	GNU Octave for the extraction of acoustic parameters such as pitch, intensity and formant bandwidths Random Forest (300 trees) for the identification of four mental disorders	Forests with 300 trees attained the greatest discrimination performance (accuracy of 75.27% and kappa index of 0.6908). Specifically, depression group got 0.713 of sensitivity, 0.925 of specificity, and 0.940 for area under ROC curve. Schizophrenic group: 0.700 of sensitivity, 0.913 of specificity, and 0.929 for area under ROC curve. Bipolar disorder: 0.830 for sensitivity, 0.952 for specificity, and 0.966 for area under ROC curve Generalized anxiety disorder: 0.920 for sensitivity, 0.943 for specificity, and 0.985 for area under ROC curve. Control group: 0.713 of sensitivity, 0.925 of specificity, and 0.940 for area under ROC curve.
De Boer et al., 2023	Estabilish the diagnostic potential of specific speech parameters in a sample of patients with a schizophrenia-spectrum disorder and analyze the ability of acoustic analyses in differentiating between patients who experience predominantly positive versus negative psychotic symptoms.	*N* = 284 −142 patients with a schizophrenia-spectrum disorder −142 matched controls	PANSS Semi-structured interviews OpenSMILE for speech analysis	OpenSMILE for the acoustic feature extraction (frequency, energy, spectral and temporal) using the extended Geneva Minimalistic Acoustic Parameter Set (eGeMAPS) ML classification for schizophrenia	The machine-learning achieved an accuracy of 86.2% (AUC of 0.92) in identifying patients with a schizophrenia-spectrum disorder and healthy controls. Moreover, it allowed to classify patients with predominantly positive or negative symptoms with an accuracy of 74.2% (AUC–ROC of 0.76). 10 acoustic parameters had the highest importance scores in the final model (*p* value <0.001).

## 3 Results

Twelve empirical studies were found through literature search (see Table 1), including case–control, cross-sectional, longitudinal and ML-based classification designs. The majority of studies were about a specific psychiatric disorder (5 were conducted on schizophrenic patients and 5 on depressed ones); one of them focused on two disorders (depression and PTSD) and another one considered four diagnostic categories (major depressive disorder, bipolar disorder, schizophrenia or generalized anxiety disorder). Generally, they used vocal acoustic analysis and machine learning to analyze several categories of acoustic features; four studies instead employed speech emotion recognition model.

### 3.1 Schizophrenia

A study of Martínez-Sánchez et al. (2015), conducted on 45 patients with schizophrenia and 35 healthy controls, showed that schizophrenic patients generally present less pitch variability in speech, make more pauses and show a significantly lower voice intensity than controls: they therefore exhibited a prosodic and melodically flatter speech. De Boer et al. (2023) found a top 10 of acoustic parameters that allowed to distinguish 142 patients with a schizophrenia-spectrum disorder from 142 matched controls. In particular, patients were classified using temporal features, such as a fragmented speech and longer pauses, and spectral ones, such as a reduced mean spectral slope and spectral flux variation, which, respectively, indicate a more tensed and monotonous voice in the patients. Moreover, some of these speech parameters can be useful to identify subjects with predominant positive or negative symptoms in schizophrenia-spectrum disorders: subjects with positive symptoms presented less variation in jitter (indicating rough voice), reduced variation in vowel frequency and a smaller F1 and F2 formant bandwidth (indicating breathiness). Other studies employed acoustic parameters to identify negative symptoms of schizophrenia; for example, Tahir et al. (2019) analyzed non-verbal speech signals (e.g., prosodic and conversational cues) as objective measures of negative symptoms of schizophrenia, obtaining significant correlations between these features and specific indicators of the 16-item Negative Symptom Assessment (NSA-16)—a semi-structured interview used to measure the severity of negative symptoms. A promising automatic procedure to detect the affective flattening, which is a typical negative symptoms of schizophrenia, was proposed in a study of He et al. (2020), conducted on 56 subjects (28 patients and 28 healthy controls); it was based on three speech characteristics: the symmetric spectral difference level (SSDL), useful to study spectral differences related to emotional richness, the quantization error and vector angle (QEVA), which reflect the variations in tone, and the standard dynamic volume value (SDVV), representing the modulation of speech intensity. The most promising feature is the SDVV feature: it achieved an accuracy of more than 85% in recognizing schizophrenic patients’ speech in each emotional state (especially the “afraid” and “happy” states); however, the combination of these acoustic features achieved a higher level of accuracy (98.2%) in detecting schizophrenia (He et al., 2020).

### 3.2 Depression

Patil and Wadhai (2021) extracted several acoustic features from the spontaneous speech of 129 participants (54 depressed patients and 75 controls) using different classifiers; results demonstrated that some of these parameters, such as MFCC, pitch, jitter (a measure of frequency instability), shimmer (related to amplitude variation in voice) and energy, can be successfully used as reliable biomarkers for depression assessment. Rejaibi et al. (2022) proposed an MFCC-based Recurrent Neural Network to detect depression and to assess its severity level from speech: low-level and high-level audio features are extracted from 189 audio recordings (56 patients with depression and 133 healthy controls) to predict the 24 scores of the Patient Health Questionnaire (PHQ-8). They showed that MFCC-based high-level features provided significant information related to depression. Stolar et al. (2018) analyzed adolescent depression detection from a clinical database of 63 adolescents (29 depressed patients and 34 controls) interacting with a parent. Many spectral parameters were investigated (i.e., flux, centroid, formants and power spectral density) to identify depression; however, the optimized spectral roll-off set, which represents the frequency-energy relationship, proved to be the most effective compared to other spectral features to detect depression. In a study of Lee et al. (2021) conducted on 61 elderly Koreans with major depressive disorder (MDD), a gender difference was found in acoustic features related to depression: acoustic characteristics with considerable discriminatory performances concerned prosody in females and speech spectrum and energy in males; in particular, males with MDD presented lower loudness compared to controls.

### 3.3 Other mental disorders

Two studies considered more diagnostic disorders. Specifically, Scherer et al. (2015) examinated an automatic unsupervised machine learning based approach to detect vowel space, a measure of frequency range related to vowel articulation, extracted from the conversational speech of 256 individuals (47 depressed patients, 88 patients with PTSD, 205 no-depressed and 165 no-PTSD patients). Findings showed that subjects with depression and PTSD presented a significantly reduced vowel space. Wanderley Espinola et al. (2022) instead proposed a methodology to support the diagnosis of schizophrenia, major depressive disorder (MDD), bipolar disorder (BD), and generalized anxiety disorder using vocal acoustic analysis and machine learning. They found that some vocal characteristics are unique for a specific group whereas others are shared by different groups. For instance, an increased pitch variability and increased intensity/volume are typical in bipolar disorder; reduced pitch range occurs both in depression and schizophrenia.

### 3.4 Speech emotion recognition

Four of the analyzed studies used an emotion recognition model to evaluate mood disorders or negative symptoms of schizophrenic patients, demonstrating that it is a promising method in diagnosis of mental diseases. A commonly used open-source feature extraction toolkit for speech emotion recognition is OpenSMILE. For instance, De Boer et al. (2023) extracted four types of acoustic parameters with OpenSMILE, employing the extended Geneva Acoustic Minimalistic Parameter Set (eGeMAPS; Eyben et al., 2015): energy/amplitude, frequency, temporal and spectral features; the trained classifier achieved an accuracy of 86.2% (AUC of 0.92) in distinguishing schizophrenia-spectrum patients from controls. In Lee et al. (2021) speech data were analyzed using two emotion recognition sets, the Audio-Visual Emotion Challenge 2013 (AVEC 2013) audio baseline feature set (Valstar et al., 2013) and the extended Geneva Minimalistic Acoustic Parameter Set (eGeMAPS). Chakraborty et al. (2018) used low-level acoustic prosodic features to distinguish between 52 individuals with schizophrenia and 26 healthy subjects and accurately detect the presence and severity of negative symptoms; furthermore, results showed that the subjective valuations of NSA-16 (16 items-Negative Symptom Assessment) could be precisely predicted from the objective acoustic features extracted with OpenSMILE “emobase” set. Finally, Hansen et al. (2022) employed a Mixture-of-Experts machine learning model to recognize two emotional states (happy and sad) using three available emotional speech datasets in German and English. They demonstrated how this speech emotion recognition model allows to detect modifications in depressed patients’ speech before and after remission; specifically, depressed patients had a higher probability of sounding sad than controls, whereas the voice of patients in remission was more happy sounding compared to the period of disease.

### 3.5 Risk of bias

No formal assessment of bias risk was conducted, as this is not required for scoping reviews according to the PRISMA-ScR guidelines. However, potential biases were considered narratively. Two reviewers independently analyzed the studies, discussing any discrepancies until consensus was reached. While acknowledging that the inclusion of only recent articles in English may have introduced selection bias, the results were interpreted with caution and methodological transparency.

## 4 Discussion

A total of 12 studies that evaluate acoustic parameters from speech to detect clinical disorders were reviewed; all of them confirm results of previous works, showing that acoustic features can be valid biomarkers of mental disorders (Tahir et al., 2019; Stolar et al., 2018). Beyond supporting the validity of speech signals analysis in detecting a mental disorder, these studies also highlighted that some mental illnesses are associated with specific voice patterns and specific changes in speech prosody or spectrum.

For instance, schizophrenic patients present prosodic and melodically flatter speech, decreased spectral slope (indicating more tension in the voice) and show a significantly lower voice intensity than healthy controls (Martínez-Sánchez et al., 2015); they also show fragmented speech and make more pauses than control group. Temporal parameters proved to be very important in identifying patients and controls: for instance, reduced speech rate can be related to slower processing speed or slower articulation (Çokal et al., 2019). Furthermore, some speech parameters can be used to distinguish between patients with positive and negative symptoms in schizophrenia-spectrum disorders. Subjects with positive symptoms generally present less variation in jitter, differences in vowel quality and a lower F1 formant frequency (De Boer et al., 2023); characteristics as low-level acoustic prosodic features and three speech parameters, related, respectively, to spectral signals (SSDL), variations in speech tone (QEVA) and intensity (SDVV), allow instead to identify negative symptoms. The anomalies of affective prosody are indeed directly related to the blunting of emotional affect (Chakraborty et al., 2018); moreover, since SDVV feature is related to speech emotional fluctuation, it can help in detecting monotonous speech, which is typical of schizophrenic patients with affective flattening (He et al., 2020) These results are in line with previous research showing that schizophrenia is associated with a general lower vocal expressivity, reduced variations in vocal pitch and energy, and lower speed (Rapcan et al., 2010; Compton et al., 2018).

Also depression is related to different changes in acoustic parameters, such as MFCC, pitch, jitter, shimmer and energy (Rejaibi et al., 2022): in particular, depressed patients’ speech is characterized by higher range of jitter and lower shimmer compared to healthy controls (Patil and Wadhai, 2021); furthermore, lower voice energy can be considered a clinical manifestation of depression (Marmor et al., 2016). A study showed that acoustic features discriminating depressed patients from control group were different in males and females: spectrum and energy-related features were specific in males and prosody-related features (e.g., F0) in females; since F0 is influenced by hormonal changes occurring in females, and since estrogens are associated with a higher incidence of depression in females, it can be assumed that this feature represents the physiology of depressive disorder in females (Lee et al., 2021).

An interesting parameter associated with depression and PTSD is the significantly reduced vowel space, a measure of frequency range related to vowel articulation; this characteristic is probably due to the typical psychomotor retardation influencing motor control, that is a common symptom of Parkinson’s disease too (Scherer et al., 2015). A study has instead confirmed that depression shares some acoustic characteristics with schizophrenia, such as reduced pitch range (Wanderley Espinola et al., 2022). These results confirm the previous literature that showed how depressed patients’ speech generally presents monotonous loudness and pitch (France et al., 2000) and lower articulation rate (Cannizzaro et al., 2004; Alpert et al., 2001).

However, unlike former reviews that primarily focused on the vocal parameters of mental disorders such as schizophrenia or depression, this work broadens the scope of research by focusing specifically on studies that have employed SER systems. This represents a very innovative approach, little explored in previous reviews (Jordan et al., 2025). A part of the examined studies is indeed focused on detecting mental disorders through speech emotion recognition system: the majority of them employed OpenSMILE toolkit, which proved to be useful to extract several emotion-related acoustic parameters from participants’ speech, such as temporal, spectral and prosodic ones (De Boer et al., 2023; Lee et al., 2021). These studies consistently underline how emotional prosody can be considered an intermediary between acoustic features and specific psychiatric symptoms, especially in mood and schizophrenia spectrum disorders. For example, depressed patients generally show a more sad sounding compared to healthy controls: this reflects the anhedonia and neurovegetative symptoms which are typical of melancholic subtype of depression (Hansen et al., 2022); these findings are consistent with results of a study from Khorram et al. (2018) on 12 patients with bipolar disorder, which showed that manic states are related to more positive and activated emotions compared to depressed states. Moreover, low-level acoustic signals proved to be a significant mark of affective prosody dysfunction in schizophrenia (Chakraborty et al., 2018).

Although a quantitative synthesis was not conducted, the qualitative analysis of the data still allows us to identify differences and similarities between the studies examined. However, this work presents some limitations. First, the analyzed studies show a great heterogeneity as regards methods: in some of them participants are recorded during an interview with a psychologist (Tahir et al., 2019; Chakraborty et al., 2018); others employed text reading which elicited emotions (He et al., 2020; Martínez-Sánchez et al., 2015) or mood-inducing sentences (MIS; Lee et al., 2021). Moreover several features extraction methods, such as OpenSMILE (Chakraborty et al., 2018; Lee et al., 2021; De Boer et al., 2023) or other open software for speech analysis (Patil and Wadhai, 2021; Wanderley Espinola et al., 2022; Scherer et al., 2015) were employed, and different classifiers, like a simple decision tree (He et al., 2020) or Random Forest algorithm, Support Vector Machine (SVM), Gaussian Mixture Model (GMM; Patil and Wadhai, 2021; Tahir et al., 2019; Chakraborty et al., 2018) have been applied. Finally, of all studies, only one was longitudinal, and only two considered more diagnostic categories.

New developments in active learning offer a promising strategy to address the two most important challenges raised in this work related to speech emotion recognition (SER) – limited labeled data availability and class imbalance. Recent studies have proposed several effective SER frameworks that not only reduce labeling costs but also improve emotion recognition accuracy. Li et al. (2023), for example, introduced the AFTER framework that combines iterative sample selection with adaptive fine-tuning; furthermore, Han et al. (2013) showed that active learning can also be effectively used in dimensional emotion recognition, demonstrating that selecting more uncertain samples allows maintaining performance similar to that of fully supervised models while using 12% less labeled data in offline (pool-based) systems and 6–11% less labeled data in online (stream-based) systems, respectively.

## 5 Conclusion

Findings support the utilization of speech analysis to detect several psychiatric disorders: it is accessible, non-invasive and can provide earlier diagnosis along with higher treatment personalization. All the studies analyzed indeed confirm that acoustic features can be used as valid biomarkers of mental disorders. Furthermore, some of them underlined that some mental diseases are associated with specific alterations in speech prosody or spectrum: specifically, depressive speech is characterized by monotonous loudness and pitch, lower speech rate and a sadder sounding. MFCC based high-level features offer significant information about depression. Some of these features occur in schizophrenia too, in particular in schizophrenic patients with affective flattening: they generally show prosodic and melodically flatter speech, a lower voice intensity, fragmented speech, and make more pauses compared to healthy controls. Although many of the findings confirm those of previous studies on the acoustic features of mental disorders, the innovative aspect of this review is to offer an updated analysis not only of the diagnostic value of vocal parameters in general, but also of SER systems. The integration of these two lines of research represents a promising perspective for early diagnosis in psychiatry through various speech biomarkers. Future studies should work on larger samples and evaluate clinical implications of these procedures in longitudinal studies; moreover, trans-diagnostic studies could allow to better identify disorders-specific acoustic features, as well as improve generalization.

## Data Availability

The original contributions presented in the study are included in the article, further requests for information may be addressed to the corresponding author.

## References

[ref1] Alghowinem S. Goecke R. Wagner M. Epps J. Breakspear M. Parker G. (2013). “Detecting depression: a comparison between spontaneous and read speech.” In *2013 IEEE International Conference on Acoustics, Speech and Signal Processing* (pp. 7547–7551). IEEE.

[ref2] Alpert M. Pouget E. R. Silva R. R. (2001). Reflections of depression in acoustic measures of the patient’s speech. J. Affect. Disord. 66, 59–69. doi: 10.1016/S0165-0327(00)00335-9, PMID: 11532533

[ref3] Bhavya S. Nayak D. S. Dmello R. C. Nayak A. Bangera S. S. (2023), “Machine learning applied to speech emotion analysis for depression recognition.” In *2023 international conference for advancement in technology (ICONAT)* (pp. 1–5). IEEE.

[ref4] Cannizzaro M. Harel B. Reilly N. Chappell P. Snyder P. J. (2004). Voice acoustical measurement of the severity of major depression. Brain Cogn. 56, 30–35. doi: 10.1016/j.bandc.2004.05.003, PMID: 15380873

[ref5] Cansel N. Alcin Ö. F. Yılmaz Ö. F. Ari A. Akan M. Ucuz İ. (2023). A new artificial intelligence-based clinical decision support system for diagnosis of major psychiatric diseases based on voice analysis. Psychiatr. Danub. 35, 489–499. doi: 10.24869/psyd.2023.489, PMID: 37992093

[ref6] Chakraborty D. Yang Z. Tahir Y. Maszczyk T. Dauwels J., (2018). “Prediction of negative symptoms of schizophrenia from emotion related low-level speech signals.” In *2018 IEEE International Conference on Acoustics, Speech and Signal Processing (ICASSP)* (pp. 6024–6028). IEEE.

[ref7] Chen Z. S. Galatzer-Levy I. R. Bigio B. Nasca C. Zhang Y. (2022). Modern views of machine learning for precision psychiatry. Patterns 3:100602. doi: 10.1016/j.patter.2022.100602, PMID: 36419447 PMC9676543

[ref8] Çokal D. Zimmerer V. Turkington D. Ferrier N. Varley R. Watson S. . (2019). Disturbo del ritmo del pensiero: modelli di pausa del linguaggio nella schizofrenia, con e senza disturbo formale del pensiero. PLoS One 14:e0217404. doi: 10.1371/journal.pone.0217404 31150442 PMC6544238

[ref9] Compton M. T. Lunden A. Cleary S. D. Pauselli L. Alolayan Y. Halpern B. . (2018). The aprosody of schizophrenia: computationally derived acoustic phonetic underpinnings of monotone speech. Schizophr. Res. 197, 392–399. doi: 10.1016/j.schres.2018.01.007, PMID: 29449060 PMC6087691

[ref10] Cummins N. Scherer S. Krajewski J. Schnieder S. Epps J. Quatieri T. F. (2015). A review of depression and suicide risk assessment using speech analysis. Speech Comm. 71, 10–49. doi: 10.1016/j.specom.2015.03.004

[ref11] De Boer J. N. Voppel A. E. Brederoo S. G. Schnack H. G. Truong K. P. Wijnen F. N. K. . (2023). Acoustic speech markers for schizophrenia-spectrum disorders: a diagnostic and symptom-recognition tool. Psychol. Med. 53, 1302–1312. doi: 10.1017/S0033291721002804, PMID: 34344490 PMC10009369

[ref12] Eyben F. Scherer K. R. Schuller B. W. Sundberg J. André E. Busso C. . (2015). The Geneva minimalistic acoustic parameter set (GeMAPS) for voice research and affective computing. IEEE Trans. Affect. Comput. 7, 190–202. doi: 10.1109/TAFFC.2015.2457417

[ref13] France D. J. Shiavi R. G. Silverman S. Silverman M. Wilkes M. (2000). Acoustical properties of speech as indicators of depression and suicidal risk. IEEE Trans. Biomed. Eng. 47, 829–837. doi: 10.1109/10.846676, PMID: 10916253

[ref14] Han W. Li H. Ruan H. Ma L. Sun J. Schuller B. W. (2013). “Active learning for dimensional speech emotion recognition” in Proceedings of Interspeech. eds. Bimbot F. Cerisara C. Fougeron C. Gravier G. Lamel L. Pellegrino F. . Lyon, France: International Speech Communication Association (ISCA). 2841–2845.

[ref15] Hansen L. Zhang Y. P. Wolf D. Sechidis K. Ladegaard N. Fusaroli R. (2022). A generalizable speech emotion recognition model reveals depression and remission. Acta Psychiatr. Scand. 145, 186–199. doi: 10.1111/acps.13388, PMID: 34850386

[ref16] Harati S. Crowell A. Mayberg H. Nemati S. (2018). “Depression severity classification from speech emotion.” In *2018 40th Annual International Conference of the IEEE Engineering in Medicine and Biology Society (EMBC)* (pp. 5763–5766). IEEE. 10.1109/EMBC.2018.8513610 30441645

[ref17] Hashem A. Arif M. Alghamdi M. (2023). Speech emotion recognition approaches: a systematic review. Speech Comm. 154:102974. doi: 10.1016/j.specom.2023.102974

[ref18] Hashim N. W. Wilkes M. Salomon R. Meggs J. France D. J. (2017). Evaluation of voice acoustics as predictors of clinical depression scores. J. Voice 31, 256.e1–256.e6. doi: 10.1016/j.jvoice.2016.06.006, PMID: 27473933

[ref19] He F. Fu J. He L. Li Y. Xiong X. (2020). Automatic detection of negative symptoms in schizophrenia via acoustically measured features associated with affective flattening. IEEE Trans. Autom. Sci. Eng. 18, 586–602. doi: 10.1109/TASE.2020.3022037

[ref20] Jiang H. Hu B. Liu Z. Wang G. Zhang L. Li X. . (2018). Detecting depression using an ensemble logistic regression model based on multiple speech features. Comput. Math. Methods Med. 2018, 1–9. doi: 10.1155/2018/6508319, PMID: 30344616 PMC6174772

[ref21] Jordan E. Terrisse R. Lucarini V. Alrahabi M. Krebs M. O. Desclés J. . (2025). Speech emotion recognition in mental health: systematic review of voice-based applications. JMIR Mental Health 12:e74260. doi: 10.2196/74260, PMID: 41027025 PMC12521853

[ref22] Kerkeni L. Serrestou Y. Mbarki M. Raoof K. Mahjoub M. A. Cleder C. (2019). “Automatic speech emotion recognition using machine learning” in Social media and machine learning [working title]. ed. Karray F.. London, UK: IntechOpen.

[ref23] Khorram S. Jaiswal M. Gideon J. McInnis M. Provost E. M. (2018). The priori emotion dataset: linking mood to emotion detected in-the-wild. arXiv. doi: 10.48550/arXiv.1806.10658

[ref24] Koops S. Brederoo S. G. de Boer J. N. Nadema F. G. Voppel A. E. Sommer I. E. (2023). Speech as a biomarker for depression. CNS & Neurolog. Disorders 22, 152–160. doi: 10.2174/1871527320666211213125847, PMID: 34961469

[ref25] Lalitha S. Gupta D. Zakariah M. Alotaibi Y. A. (2021). Mental illness disorder diagnosis using emotion variation detection from continuous English speech. Comput. Mater. Contin. 69, 3217–3238. doi: 10.32604/cmc.2021.018406

[ref26] Lalitha S. Tripathi S. (2016). “Emotion detection using perceptual based speech features.” In *2016 IEEE annual India conference (INDICON)* (pp. 1–5). IEEE.

[ref27] Lee S. Suh S. W. Kim T. Kim K. Lee K. H. Lee J. R. . (2021). Screening major depressive disorder using vocal acoustic features in the elderly by sex. J. Affect. Disord. 291, 15–23. doi: 10.1016/j.jad.2021.04.098, PMID: 34022551

[ref28] Li D. Wang Y. Funakoshi K. Okumura M. (2023). “After: active learning based fine-tuning framework for speech emotion recognition.” In *2023 IEEE Automatic Speech Recognition and Understanding Workshop (ASRU)* (pp. 1–8). IEEE.

[ref29] Low D. M. Bentley K. H. Ghosh S. S. (2020). Automated assessment of psychiatric disorders using speech: a systematic review. Laryngoscope Investigative Otolaryngol. 5, 96–116. doi: 10.1002/lio2.354, PMID: 32128436 PMC7042657

[ref30] Manchia M. Pisanu C. Squassina A. Carpiniello B. (2020). Challenges and future prospects of precision medicine in psychiatry. Pharmacogenomics Personalized Med. 13, 127–140. doi: 10.2147/PGPM.S198225, PMID: 32425581 PMC7186890

[ref31] Mar L. L. Pa W. P. (2019). Depression detection from speech emotion recognition (Doctoral dissertation, MERAL Portal).

[ref32] Marmor S. Horvath K. J. Lim K. O. Misono S. (2016). Voice problems and depression among adults in the U nited S tates. Laryngoscope 126, 1859–1864. doi: 10.1002/lary.25819, PMID: 26691195 PMC4916046

[ref33] Martínez-Sánchez F. Muela-Martínez J. A. Cortés-Soto P. Meilán J. J. G. Ferrándiz J. A. V. Caparrós A. E. . (2015). Can the acoustic analysis of expressive prosody discriminate schizophrenia? Span. J. Psychol. 18:E86. doi: 10.1017/sjp.2015.85 26522128

[ref34] Pan W. Flint J. Shenhav L. Liu T. Liu M. Hu B. . (2019). Re-examining the robustness of voice features in predicting depression: compared with baseline of confounders. PLoS One 14:e0218172. doi: 10.1371/journal.pone.0218172, PMID: 31220113 PMC6586278

[ref35] Parola A. Simonsen A. Bliksted V. Fusaroli R. (2018). T138. acoustic patterns in schizophrenia: a systematic review and meta-analysis. Schizophr. Bull. 44:S169. doi: 10.1093/schbul/sby016.415 31839552

[ref36] Pathan H. B. Preeth S. Bhavsingh M. (2023). Revolutionizing PTSD detection and emotion recognition through novel speech-based machine and deep learning algorithms. Front. Collaborative Res. 1, 35–44.

[ref37] Patil M. Wadhai V. (2021). “Selection of classifiers for depression detection using acoustic features.” In *2021 International Conference on Computational Intelligence and Computing Applications (ICCICA)* (pp. 1–4). IEEE.

[ref38] Rapcan V. D’Arcy S. Yeap S. Afzal N. Thakore J. Reilly R. B. (2010). Acoustic and temporal analysis of speech: a potential biomarker for schizophrenia. Med. Eng. Phys. 32, 1074–1079. doi: 10.1016/j.medengphy.2010.07.013, PMID: 20692864

[ref39] Rejaibi E. Komaty A. Meriaudeau F. Agrebi S. Othmani A. (2022). MFCC-based recurrent neural network for automatic clinical depression recognition and assessment from speech. Biomed. Signal Process. Control. 71:103107. doi: 10.1016/j.bspc.2021.103107

[ref40] Scherer S. Lucas G. M. Gratch J. Rizzo A. S. Morency L. P. (2015). Self-reported symptoms of depression and PTSD are associated with reduced vowel space in screening interviews. IEEE Trans. Affect. Comput. 7, 59–73. doi: 10.1109/TAFFC.2015.2440264

[ref41] Siena F. L. Vernon M. Watts P. Byrom B. Crundall D. Breedon P. (2020). Proof-of-concept study: a mobile application to derive clinical outcome measures from expression and speech for mental health status evaluation. J. Med. Syst. 44:209. doi: 10.1007/s10916-020-01671-x, PMID: 33175234 PMC7658062

[ref42] Stasak B. Epps J. Cummins N. Goecke R. (2016). “An investigation of emotional speech in depression classification” in Proceedings of Interspeech. eds. Nelson M. Hynek H. Tony H.. San Francisco, USA: International Speech Communication Association (ISCA). 485–489.

[ref43] Stolar M. N. Lech M. Stolar S. J. Allen N. B. (2018). Detection of adolescent depression from speech using optimised spectral roll-off parameters. Biom. J. 2, 2574–1241. doi: 10.26717/BJSTR.2018.05.001156

[ref44] Tahir Y. Yang Z. Chakraborty D. Thalmann N. Thalmann D. Maniam Y. . (2019). Non-verbal speech cues as objective measures for negative symptoms in patients with schizophrenia. PLoS One 14:e0214314. doi: 10.1371/journal.pone.0214314, PMID: 30964869 PMC6456189

[ref45] Teixeira F. L. Costa M. R. E. Abreu J. P. Cabral M. Soares S. P. Teixeira J. P. (2023). A narrative review of speech and EEG features for schizophrenia detection: progress and challenges. Bioengineering 10:493. doi: 10.3390/bioengineering10040493, PMID: 37106680 PMC10135748

[ref46] Tricco A. C. Lillie E. Zarin W. O’Brien K. K. Colquhoun H. Levac D. . (2018). PRISMA extension for scoping reviews (PRISMA-ScR): checklist and explanation. Ann. Intern. Med. 169, 467–473. doi: 10.7326/M18-0850 30178033

[ref47] Valstar M. Schuller B. Smith K. Eyben F., (2013). “Avec 2013: the continuous audio/visual emotion and depression recognition challenge.” In *Proceedings of the 3rd ACM international workshop on Audio/visual emotion challenge*. Barcelona, Spain: Proceedings published by ACM. (pp. 3–10).

[ref48] Wanderley Espinola C. Gomes J. C. Mônica Silva Pereira J. dos Santos W. P. (2022). Detection of major depressive disorder, bipolar disorder, schizophrenia and generalized anxiety disorder using vocal acoustic analysis and machine learning: an exploratory study. Res. Biomedical Eng. 38, 813–829. doi: 10.1007/s42600-022-00222-2

[ref49] Wang X. Wang M. Qi W. Su W. Wang X. Zhou H. (2021), “A novel end-to-end speech emotion recognition network with stacked transformer layers.” In *ICASSP 2021–2021 IEEE International Conference on Acoustics, Speech and Signal Processing (ICASSP)* (pp. 6289–6293). IEEE.

[ref51] Zhang Y. Folarin A. A. Dineley J. Conde P. de Angel V. Sun S. . (2024). Identifying depression-related topics in smartphone-collected free-response speech recordings using an automatic speech recognition system and a deep learning topic model. J. Affect. Disord. 355, 40–49. doi: 10.1016/j.jad.2024.03.106, PMID: 38552911

