# Supplementary material for: Speech analysis and speech emotion recognition in mental disease: a scoping review
Source: Front Psychol. 2025 Nov 6;16:1645860. doi: 10.3389/fpsyg.2025.1645860 (PMC12629935; doi:10.3389/fpsyg.2025.1645860)
Supplement: Supplementary file 1 [file Data_Sheet_1.pdf]

PRISMA

PRISMA 2020 Checklist

Section and Topic	Item #	Checklist item	Location where item is reported
**TITLE**			
Title	1	Speech analysis and speech emotion recognition in mental disease: a systematic review	Title
**ABSTRACT**			
Abstract	2	Background: Mental disorders have a significant impact on many areas of people's life, particularly on affective regulation; thus there is a growing need to find disease-specific biomarkers to improve early diagnosis. Recently, machine learning technology using speech analysis proved to be a promising field that could aid mental health assessments. Furthermore, as prosodic expressions of emotions are altered in many psychiatric conditions, some studies successfully employed a speech emotion recognition model (SER) to identify mental diseases. The aim of this paper is to discuss the utilization of speech analysis in diagnosis of mental disorders, with a focus on studies using SER system to detect mental illness.<br>Method: We searched PubMed, Scopus and Google Scholar for papers published from 2014 to 2024. We conducted a preliminary search, which revealed papers on the topic. Finally, 12 studies met the inclusion criteria and were included in the review.<br>Results: Findings confirmed the efficacy of speech analysis in distinguishing between patients from healthy subjects; moreover, the examined studies underlined that some mental illnesses are associated with specific voice patterns. Furthermore, results from studies employing speech emotion recognition system to detect mental disorders showed that emotions can be successfully used as an intermediary step for mental diseases detection, particularly for mood disorders.<br>Conclusions: These findings support the implementing of speech signals analysis in mental health assessment: it is an accessible and non-invasive method which can provide earlier diagnosis and a higher treatment personalization.	Abstract
**INTRODUCTION**			
Rationale	3	A psychiatric disorder is a mental or behavioural pattern that influences emotional regulation, behaviour and cognition, causing a significant impairment in several areas of people's life, such as the functioning capacity at work and with their families (Lalitha et al., 2021). In recent years, especially during the Covid-19 pandemic, there has been a significant increase in people affected by a mental disorder, with a consequent high impact on emotional life and affective regulation: about 970 million people in the world are currently suffering from a mental dis¬order and the number is expected to grow in the future (Cansel et al., 2023). To now, there is still a lack of biomarkers and individualized treatment guidelines for mental illnesses (Chen et al., 2022). Machine learning technology seems to be a promising field in mental health assessments: it may indeed be useful in screening of at-risk patients, improve the detection of disorder-specific fea-tures, allow to plan more efficient treatments and enable more real-time monitoring of psychiatric disorders (Low et al., 2020; Siena et al. 2020). In particular, the language can be considered as a window into the mind (Koops et al., 2023): people convey emotions, thoughts and motivations through speech (Zhang et al., 2024). If the speech content is easily masked by people, features such as speed, energy and pitch variation in speech can't be controlled. Many studies have demonstrated that acoustic parameters can be used as valid biomarkers for the early diagnosis of mental disorders (Pan et al. 2019; Cummins et al., 2015). Negative emotions such as sadness, anger and fear are indicator of mental disorders (Lalitha and Tripathi, 2016): for this reason, another promising approach to diagnosis of mental health conditions comes from Speech Emotion Recognition (SER), a system which provides an extraction of the speakers' emotional states from their speech signals (Kerkeni et al., 2019). It has been employed in detecting different mental illnesses, such as post-traumatic stress disorder (PTSD) (Pathan et al., 2023) and depression (Mar & Pa, 2019).	Introduction
Objectives	4	The aim of this work is to define the state of literature on acoustic features used as objective indicators for the diagnosis of mental disorders; in particular, a focus on some studies using speech emotion recognition system (SER) to detect mental illness will be presented, in order to confirm the effectiveness of this approach in mental health assessments.	Introduction
**METHODS**			
Eligibility criteria	5	In this systematic review, the eligibility criteria were defined to ensure the consistency and relevance of the included studies. We included studies focusing exclusively on detection of mental disorders through speech analysis and speech emotion recognition system (SER) in order to show that acoustic features can be valid biomarkers of mental disorders. Articles were included in the review according to the following inclusion criteria: English language, only RCT studies, studies involving clinical populations with mental disorders, studies that involved quantitative and/or qualitative assessments of the variables considered. Books, meta-analyses, and reviews were excluded; non-RCT studies and studies that did not involve quantitative and/or qualitative assessments of the variables were also excluded.	Data extraction

PRISMA

PRISMA 2020 Checklist

Section and Topic	Item #	Checklist item	Location where item is reported
Information sources	6	This search was conducted in accordance with the Preferred Reporting Items for Systematic Reviews and Meta-Analyses (PRISMA) guidelines. Google Scholar, PubMed, and Scopus databases were used to identify relevant studies.	Material and methods
Search strategy	7	Exhaustive searches were carried out in the PubMed, Google Scholar and Scopus databases. Studies exploring acoustic parameters as objective measures of mental disorders were collected from 2014 to 2024. To identify relevant articles, the search was performed within article titles, abstracts, and keywords using the following keywords: "Speech analysis OR speech emotion recognition OR acoustic analysis OR acoustic features AND mental disorders AND schizophrenia AND depression AND bipolar disorder".	Information sources and search strategy
Selection process	8	The duplicates of articles detected using literature search were remove by Zotero literature management program. A reviewer selected by titles and abstracts for suitable studies. Studies meeting all criteria were used in the review. After the screening of titles and abstract, based on default inclusion/esclusion criteria adjusted to the systematic review's objectives, the authors carefully checked the validity of each study through the examination of the abstracts or, if needed, the full articles. This meticulous process secured that only suitable studies were included in the systematic review. We found 15.854 articles. Of these, 854 were removed before screening since they were duplicates. At the first screening conducted by title and abstract, 14.962 studies were excluded. After the second screening conducted by full-text examination of 38 papers, 26 articles were excluded because they were reviews, metanalysis, not specific, irrelevant for the topic, because full text was not available or because the trial did not present a control group. Finally, 12 studies met the inclusion criteria and were included in the review.	Data synthesis
Data collection process	9	Data collection for this review was planned and executed to secure the completeness and accuracy of the information. After researching in PubMed, Google Scholar and Scopus databases up to November 2024, only data from studies in English language and published within the last 10 year were included, in order to ensure the relevance of the collection. First, reviewers separately examined the articles, using default inclusion/esclusion criteria, adjusted to the systematic review's objectives. Then, they carefully checked the validity of each study through the accurate examination of the abstracts or, if needed, the full articles. At this stage, primary data were derived following a default procedure, in particular collecting information about population, methods, results and types of studies. No automated tools were employed in data collection, so any mismatches between reviewers' data extractions were solved through discussion or, if needed, involving a third reviewer.	Materials and methods
Data items	10a	In our systematic review, we identified and defined the following primary outcomes for which data were collected: confirmation of the diagnostic value of acoustic parameters in detection of mental disorders; employment of speech emotion recognition system to identifly mental diseases.	Materials and methods
	10b	We also collected data on the following variables: participant characteristics such as age, gender, diagnosis of mental disorder, as well as details of the methods of speech analysis.	Materials and methods
Study risk of bias assessment	11	In order to determine the risk of bias in this systematic review, a critical approach coming from the reviewers' judgment was used. Each study was carefully analysed in search of potential bias, for example choice of participants, study conduct, exhaustiveness in collecting data, selection of reported results. The reviewers worked on their own to determine the risk of bias, and then compared their assessing for each study in the review. In case of mismatch, the reviewers discussed in detail to find agreement. If necessary, a third expert reviewer was consulted to resolve complex issues.	Risk of bias
Effect measures	12	The included studies measured the accuracy of different speech analysis methods in distinguishing patients from healthy controls. They also examinated specific voice patterns and specific changes in speech associated with mental disorders. Moreover, some of them evaluated the efficacy of SER model in detecting specific mental disorders, providing a baseline for emotional speech processing.	Results
Synthesis methods	13a	The selection of eligible studies for synthesis was based on predefined inclusion and exclusion criteria. Studies were tabulated and compared based on types of acoustic parameters extracted from speech analysis, including speech emotion recognition models. Key study information was summarized in a descriptive table (Table 1), detailing the study aim, sample, materials and methods and results.	Data extraction
	13b	Since the selected studies gave complete and usable data for qualitative synthesis, no data conversions or handling of missing summary statistics were required.	
	13c	The results of each study were presented in tabular form (Table 1), summarizing the characteristics of the included studies. Moreover, a	Materials

Section and Topic	Item #	Checklist item	Location where item is reported
		PRISMA flow diagram (Figure 1) was used to illustrate the study selection process.	and methods
	13d	Qualitative descriptive synthesis was conducted: in particular, studies were compared in terms of methodology and accuracy in detecting mental diseases through speech features analysis. A meta-analysis was not performed as the variability among studies did not allow for quantitative data aggregation.	
	13e	No formal statistical methods were applied due to the heterogeneity in study designs, methods and results. Differences were instead analyzed narratively by comparing study characteristics and methods.	Material and methods and Results
	13f	No sensitivity analyses were performed, as the review is qualitative.	
Reporting bias assessment	14	Since this review did not include a meta-analysis, no formal assessment of reporting bias was conducted. Anyway, bias in publication was reduced to the minimum through the research of different databases and the application of default inclusion criteria which secured a comprehensive selection of studies.	Risk of bias
Certainty assessment	15	No formal certainty assessment was performed. Confidence in the findings was supported by applying rigorous eligibility criteria and qualitatively comparing study designs, methods and outcomes.	Risk of bias
**RESULTS**			
Study selection	16a	We found 15.854 articles. Of these, 854 were removed before screening since they were duplicates. At the first screening conducted by title and abstract, 14.962 studies were excluded. After the second screening conducted by full-text examination of 38 papers, 26 articles were excluded because they were reviews, metanalysis, not specific, irrelevant for the topic, because full text was not available or because the trial did not present a control group. Finally, 12 studies met the inclusion criteria and were included in the review. The study selection process is detailed in the PRISMA flow diagram (Figure 1).	Data synthesis
	16b	After full-text review, 26 studies were excluded because they were reviews, metanalysis, not specific, irrelevant for the topic, because full text was not available or because the trial did not present a control group.	Data synthesis
Study characteristics	17	1. Martinez-Sanchez et al., 2015- A cross-sectional study evaluating a semi-automatic method's ability to quantifying the levels of expressive prosody deficits in schizophrenia, distinguishing between patient and control groups.<br>2. Scherer et al., 2015-A cross-sectional study exploring vowel space, a measure of frequency range, extracted from conversational speech and its relationship to self-reported symptoms of depression and post-traumatic stress disorder (PTSD)<br>3. Chakraborty et al., 2018- A cross-sectional study evaluating the efficacy of acoustic features related to emotion (low-level speech signals) in the distinction of patients with schizophrenia from healthy individuals.<br>4. Stolar et al., 2018- A cross-sectional analyzing adolescent depression detection from a clinical database of 63 adolescents interacting with a parent.<br>5. Tahir et al., 2019- A cross-sectional study exploring non-verbal speech cues as objective measures of negative symptoms of schizophrenia.<br>6. He et al., 2020- A cross-sectional study evaluating an automatic system for detecting the negative symptoms of patients with schizophrenia based on speech signal processing.<br>7. Lee et al., 2021- A cross-sectional study detecting major depressive disorder using vocal acoustic features of elderly people, for males and females.<br>8. Patil & Wadhai, 2021- A cross-sectional study which evaluates depression using acoustic features extracted from the spontaneous speech samples of the volunteers.	Results

Section and Topic	Item #	Checklist item	Location where item is reported
		9. Hansen et al., 2022-A longitudinal study analyzing a method to evaluate depression and remission from acoustic speech.<br>10. Rejaibi et al., 2022- a cross- sectional study identifying depression from speech using a deep Recurrent Neural Network-based framework.<br>11. Wanderley Espinola et al., 2022- A cross-sectional study evaluating a methodology to support the diagnosis of schizophrenia, major depressive disorder, bipolar disorder, and generalized anxiety disorder using vocal acoustic analysis and machine learning.<br>12. De Boer et al., 2023- A cross-sectional study analyzing the diagnostic potential of specific speech parameters in patients with a schizophrenia-spectrum disorder differentiating between patients with predominant positive versus negative psychotic symptoms.	
Risk of bias in studies	18	A formal risk of bias assessment was not conducted, but potential biases were considered narratively. Selection bias was minimized through predefined eligibility criteria. However, the inclusion of only recent English-language publications could contribute to reporting bias. Despite these limitations, study quality was carefully evaluated, and findings were interpreted with caution.	Results
Results of individual studies	19	The results of each included study are summarized in Table 1, which presents key findings. Where applicable, summary statistics and effect estimates are reported. Due to the heterogeneity of various studies' methods, a structured synthesis instead of a meta-analysis was performed. No pooled effect estimates or confidence intervals were calculated.	Results
Results of syntheses	20a	Studies in question were different in methods and outcome measures, so direct comparisons were difficult. Risk of bias assessment wasn't formally executed, but limitation were taken into account during results interpretation	Results
	20b	No meta-analysis or formal statistical synthesis was performed due to the variability in methods and outcome measures. Anyway, a qualitative synthesis was conducted to compare methods effectiveness.	Results
	20c	Heterogeneity in studies was examined narratively comparing their features, methods and reported findings. Subgroup analysis and meta-regressions were not conducted.	Results
	20d	Since the review focused on qualitative synthesis rather than statistical modeling, no sensitivity analyses were executed. Findings were interpreted with caution, considering study limitations.	Results
Reporting biases	21	No formal assessment of reporting bias was performed. However, attempts have been made to minimize bias by searching different databases and applying predefined eligibility criteria.	Results
Certainty of evidence	22	Certainty assessment wasn't formally executed. Confidence in results was supported by precision of methods in selecting studies. Anyway, variability in methods, sample sizes and outcome measures influences the general certainty of the evidence.	Results
**DISCUSSION**			
Discussion	23a	The findings of this systematic review confirm results of previous works, showing that acoustic features can be valid biomarkers of mental disorders. Beyond confirming the validity of speech signals analysis in detecting a mental disorder, these studies also highlighted that some mental illness (i.g. schizophrenia, depression, bipolar disorder) are associated with specific voice changes in speech, in particular in prosodic, temporal and spectral parameters. Moreover, studies employing speech emotion recognition system show that emotions can be used as an intermediary step for mental diseases detection, especially for mood disorders and schizophrenia spectrum.	Discussion
	23b	This work presents some limitations; for example, the analysed studies show a great heterogeneity as regards methods: both in terms of participants' recording collection and in terms of vocal parameters extracting methods.	Discussion
	23c	Only studies published in English within the last ten years were included, which may have given publication bias and the exclusion of relevant evidence. The lack of a meta-analysis prevented quantitative synthesis, and no formal risk of bias or certainty assessments were conducted.	Discussion
	23d	Findings support the utilization of speech analysis to detect several psychiatric disorders: it is accessible, non-invasive and can provide earlier diagnosis along with higher treatment personalization. Future studies should work on larger samples and evaluate clinical implications of these procedures in longitudinal studies: moreover, trans-diagnostic studies could allow to better identify disorders-specific acoustic features, as well as improve generalization.	Discussion

PRISMA

PRISMA 2020 Checklist

Section and Topic	Item #	Checklist item	Location where item is reported
**OTHER INFORMATION**			
Registration and protocol	24a	This review hasn't been registered in PROSPERO, the International Prospective Register of Systematic Reviews, National Institute of Health Research, University of York.	
	24b	A protocol was not prepared.	
	24c	None.	
Support	25	This work was supported by the European Union - Next Generation EU under the Italian National Recovery and Resilience Plan (NRRP), Mission 4, Component 2, Investment 1.3, CUP C49J24000240004, partnership on "Telecommunications of the Future" (PE00000001 - program "RESTART").	Support
Competing interests	26	The authors declare that they have no competing interests related to this systematic review. There are no financial, personal, or professional conflicts of interest to disclose.	Conflict of interests
Availability of data, code and other materials	27	Since this review is a qualitative synthesis of published studies, no raw data, analytic code, or data extraction forms are available. There were no new data collection or analyses conducted outside the data provided in the included studies. All materials used in the review, including study details, are described in the final manuscript.	NA

From: Page MJ, McKenzie JE, Bossuyt PM, Boutron I, Hoffmann TC, Mulrow CD, et al. The PRISMA 2020 statement: an updated guideline for reporting systematic reviews. BMJ 2021;372:n71. doi: 10.1136/bmj.n71. This work is licensed under CC BY 4.0. To view a copy of this license, visit https://creativecommons.org/licenses/by/4.0/
